# Modulation of Innate Immune Responses by the Influenza A NS1 and PA-X Proteins

**DOI:** 10.3390/v10120708

**Published:** 2018-12-12

**Authors:** Aitor Nogales, Luis Martinez-Sobrido, David J. Topham, Marta L. DeDiego

**Affiliations:** 1 Department of Microbiology and Immunology, University of Rochester, Rochester, New York, NY 14642, USA; Luis_martinez@urmc.rochester.edu (L.M.-S.); David_Topham@urmc.rochester.edu (D.J.T.); 2 Centro de Investigación en Sanidad Animal (CISA)-INIA, Valdeolmos, 28130 Madrid, Spain; 3 David H. Smith Center for Vaccine Biology and Immunology, University of Rochester, Rochester, New York, NY 14642, USA; 4 Department of Molecular and Cell Biology, Centro Nacional de Biotecnología (CNB-CSIC), Campus Universidad Autónoma de Madrid, 28049 Madrid, Spain

**Keywords:** influenza a virus, NS1, PA, PA-X, interferon, host antiviral response, innate immunity, cytokines, virus–host interactions, pathogenesis, virus evolution, vaccine

## Abstract

Influenza A viruses (IAV) can infect a broad range of animal hosts, including humans. In humans, IAV causes seasonal annual epidemics and occasional pandemics, representing a serious public health and economic problem, which is most effectively prevented through vaccination. The defense mechanisms that the host innate immune system provides restrict IAV replication and infection. Consequently, to successfully replicate in interferon (IFN)-competent systems, IAV has to counteract host antiviral activities, mainly the production of IFN and the activities of IFN-induced host proteins that inhibit virus replication. The IAV multifunctional proteins PA-X and NS1 are virulence factors that modulate the innate immune response and virus pathogenicity. Notably, these two viral proteins have synergistic effects in the inhibition of host protein synthesis in infected cells, although using different mechanisms of action. Moreover, the control of innate immune responses by the IAV NS1 and PA-X proteins is subject to a balance that can determine virus pathogenesis and fitness, and recent evidence shows co-evolution of these proteins in seasonal viruses, indicating that they should be monitored for enhanced virulence. Importantly, inhibition of host gene expression by the influenza NS1 and/or PA-X proteins could be explored to develop improved live-attenuated influenza vaccines (LAIV) by modulating the ability of the virus to counteract antiviral host responses. Likewise, both viral proteins represent a reasonable target for the development of new antivirals for the control of IAV infections. In this review, we summarize the role of IAV NS1 and PA-X in controlling the antiviral response during viral infection.

## 1. Introduction

### 1.1. Influenza A Virus (IAV)’s Relevance to Human Health

IAV are members of the *Orthomyxoviridae* family of enveloped viruses, which contain an eight-segmented, single-stranded (ss), negative-sense RNA genome [1]. IAV can infect multiple hosts, including birds, pigs, dogs, horses, bats, and humans [2,3,4,5]. These viruses undergo reassortment in wild hosts, leading to the emergence of novel strains with epidemic or pandemic potential in humans. In fact, IAV is one of the main causes of respiratory infections in humans, and are responsible for seasonal epidemics each year and occasional pandemics of great consequence, such as the 1918 “Spanish flu” [2,6,7]. IAV is classified in subtypes according to the hemagglutinin (HA; H1–H18) and neuraminidase (NA; N1–N11) sequences [8], with 18 HA subtypes and 11 NA subtypes currently circulating [4,9,10,11]. In humans, the most frequent seasonal IAV circulating nowadays are the H3N2 and the H1N1 subtypes of IAV [12,13]. However, human cases of the highly pathogenic H5N1 avian IAV continue to occur throughout parts of East and Southeast Asia, the Middle East, Africa, and Europe [9,14]. In addition, outbreaks of H6, H7, H9, and H10 IAV in poultry and zoonotic infections of humans with these viral strains have been reported [9,14]. Fortunately, these antigenically novel IAV have yet to sustain human–to-human transmission, and have, therefore, failed to generate a potentially devastating human pandemic [9,14]. The first IAV pandemic in the 21st century started in 2009 with the emergence of a quadruple-reassortant swine-origin H1N1 IAV (pH1N1) [15,16], which, in less than one year, infected more than 600,000 individuals around the world, although this number is likely underestimated. Importantly, this virus is efficiently transmitted among humans and continues circulating seasonally [12,17,18]. Although comprehensive vaccination programs are in place, the global disease burden from seasonal influenza results in 1 billion infections, 3–5 million cases of severe disease, and between 250,000 and 500,000 deaths annually, according to the World Health Organization (WHO) [7,19], leading also to a significant worldwide economic impact.

### 1.2. Interferon and Inflammatory Cytokine Responses during IAV Infection

The host innate immune system restricts IAV replication [20]. When IAV infects a cell, pathogen-associated molecular patterns (PAMPs) are recognized by pattern recognition receptors (PRRs). This recognition activates signaling pathways, leading to the production of type I (IFNα and IFNβ) and III (IFNλ) interferons (IFNs) and cytokines involved in inflammatory processes [20]. IAV double-stranded (ds) or single-stranded (ss) RNAs are recognized by the membrane-associated PRR Toll-like receptors (TLRs) 3 or 7 and 8, respectively [20,21], and by the cytoplasmic PRR retinoic acid-inducible gene I (RIG-I) that detect double-stranded (ds)RNA and 5’- triphosphates of the (-) single-stranded (ss)RNA viral genome [20,22] generated during virus replication. The 5′ and 3′ ends of the influenza’s viral RNAs contain partially complementary sequences that are able to form short 5′ppp-dsRNA structures [23]. The consequence of TLRs and RIG-I detection of IAV PAMPs is the activation of transcription factors, such as IFN regulatory factors (IRF) 3 and 7, the nuclear factor kappa β (NF-κB), and the activator protein 1 (AP-1) transcription factors, that are responsible for the transcription of type I and type III IFN [20,24,25] and pro-inflammatory cytokines [26]. Secreted type I and III IFNs act in the same cell or in surrounding cells to induce the expression of IFN-stimulated genes (ISGs), many of which display antiviral activity [20,25,27]. Type I and III IFNs signal through different receptors; however, the pathways converge in the phosphorylation of the signal transducer and activator of transcription (STAT) 1 and STAT2 transcription factors [28]. Phosphorylated forms of STAT1 and STAT2 then associate with IRF9 to form a heterotrimeric ISG factor 3 (ISGF3) complex [28]. ISGF3 then translocates to the nucleus, where it binds to IFN-stimulated response elements (ISREs) sequences present in the promoter of ISGs to upregulate their transcription [28,29]. The Janus protein tyrosine kinase 1 (JAK1) and tyrosine kinase 2 (TYK2) are critical for phosphorylation and activation of STAT1 and STAT2 [30]. In addition, STAT1 is phosphorylated by IKKε during IFN signaling, and this step is critical for the IFN-inducible antiviral response [31,32].

The inflammasome is a molecular complex formed by the proteins Nucleotide oligomerization domain (NOD)-like receptor family member LRR- and Pyrin domain containing-3 (NLRP3), Apoptosis-associated Speck-like containing a caspase-recruitment domain (ASC), and pro-caspase 1 [33]. NLRP3 is expressed by myeloid cells, such as monocytes, macrophages, neutrophils, and dendritic cells [34], and also by human bronchial epithelial cells [35]. The inflammasome activation involves the sensing of the PAMPs by NLRP3. Moreover, protein flux through the viral matrix protein 2 (M2) ion channel in the trans-Golgi network triggers NLRP3 activation [36]. In addition, it has been shown that IAV PB1-F2 [37] and dsRNA [38] activate the inflammasome, which stimulates the activation of caspase-1. Active caspase-1 cleaves inactive pro-interleukin (IL)-1β and pro-IL-18 into their mature forms IL-1β and IL-18, respectively, which are released from the cell to stimulate inflammatory processes [39].

### 1.3. ISGs against IAV Infections

Many ISGs, such as myxovirus resistance (Mx), IFN-induced transmembrane (IFITM), protein kinase R (PKR), and 2’-5’ oligoadenylate synthetase (OAS)-ribonuclease L (RNAseL), limit IAV replication [20,40]. In mice, Mx1 is expressed in the nucleus and displays antiviral activity [41] by blocking viral mRNA elongation [42,43], whereas Mx2 is expressed in the cytoplasm and does not show antiviral activity. In humans, the MxA (also known as Mx1) protein is cytosolic, and has potent antiviral activity against IAV [44]. Inhibition of IAV by MxA involves binding to the viral nucleoprotein (NP) and the inhibition of viral transcription [44,45]. By contrast, the MxB (also known as Mx2) protein is present at the cytoplasmic face of nuclear pore complexes and it does not inhibit IAV infection [46].

Members of the interferon-induced transmembrane (IFITM) proteins family are able to prevent infection before a virus can traverse the lipid bilayer of the cell. At least three human IFITM proteins (IFITM1, IFITM2, and IFITM3) have been described to have antiviral activity by blocking virus–host cell membrane fusion following viral attachment and endocytosis [47,48]. Cumulative evidence indicates that IFITM1 is localized predominantly at the plasma membrane, while IFITM2 and 3 localize to endosomal and lysosomal compartments, respectively [49].

Activation of OAS by dsRNA produces poly(A) chains with 2′-5′ phosphodiester bonds [50], which bind to and activate constitutively expressed RNaseL, leading to the cleavage of viral and cellular ssRNA, resulting in the inhibition of virus replication [51]. In addition, it has been shown that some of the viral ssRNA degradation products bind to and activate RIG-I, leading to an enhanced activation of IFN transcription [22,52]. Activity of the OAS/RNase L pathway was also demonstrated in vitro against many viruses, in particular RNA viruses [53]. However, some RNA viruses, such as IAV, are hardly affected, because they express antagonist proteins (see Section 2.2).

Protein kinase R (PKR) is activated by dsRNA or by the cellular protein activator of the interferon-induced PKR (PACT), resulting in autophosphorylation and phosphorylation of cellular proteins, including the α subunit of the eukaryotic initiation factor 2 (eIF2α) [54]. This phosphorylation leads to inhibition of protein synthesis, including viral proteins, in infected cells. In addition, PKR activates the transcription factor NF-κB by phosphorylating IκB [55]. Furthermore, PKR stabilizes type I IFN mRNA, thereby ensuring robust IFN protein production [56]. It has been shown that PKR-deficient mice are more susceptible to various viral infections, including IAV, further demonstrating the remarkable antiviral activity of this ISG product [57].

## 2. The IAV Non-Structural 1 (NS1) Protein

### 2.1. NS1 Protein Introduction

**Figure 1:**
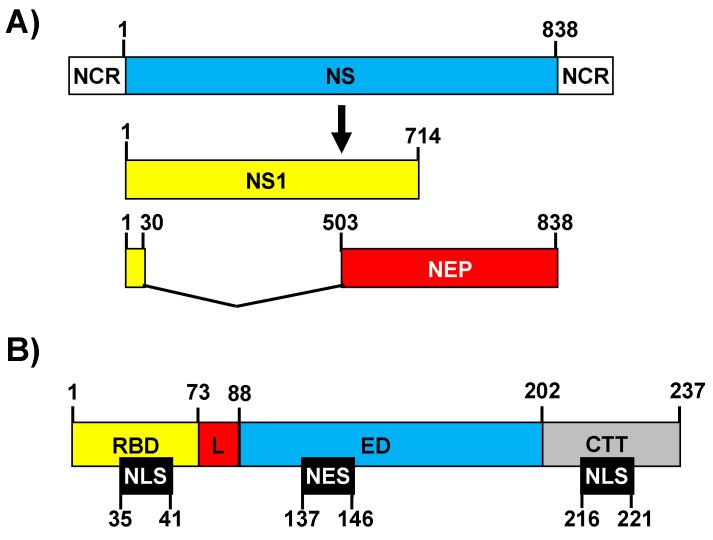
A schematic representation of an influenza A virus (IAV) non-structural (NS) segment, viral transcripts, and NS1 domains. (**A**) An IAV NS RNA segment is indicated by a blue box and non-coding regions (NCR) are indicated with white boxes. IAV NS1 and nuclear export protein (NEP) transcripts are indicated with yellow and red boxes, respectively. IAV NS1 and NEP open reading frames (ORFs) shared the first 30 nucleotides in the N-terminus. The numbers on the top of the bars represent the ORF length and nucleotide splice positions. (**B**) The NS1 protein is divided into four distinct regions: The N-terminal RNA-binding domain (RBD; amino acids 1–73, yellow), the linker sequence (L; amino acids 74–88, red), the effector domain (ED; amino acids 89–202, blue), and the C-terminal tail (CTT; amino acids 203 to the end, gray). Note that both the L and the CTT can vary in length among different IAV strains, and, although a 237 amino-acids-length NS1 has been represented, the NS1 can be 219, 230, and 237 amino acids in length. Nuclear localization and export signals (NLS and NES, respectively) are indicated with black boxes at the bottom.

The IAV genome segment 8 encodes the non-structural (NS) mRNA as a continuous primary transcript [58] from which NS1 is synthesized. In addition, alternative processing of this transcript using a weak 5′ splice site results in a less abundant spliced product encoding the nuclear export protein (NEP) [58]. This second transcript accounts for 10–15% of the NS-derived mRNA (Figure 1A) [59]. NS1 is most often a 230 amino acid protein [60]. However, mutations that either suppress the stop codon at position 231 or create a premature stop codon result in length variations [60]. For instance, from the late 1940s until the middle of the 1980s, NS1 of human IAV harbored the C-terminus, seven-amino-acids extension 231-RRNKMAD-237 [60,61]. Conversely, the NS1 protein of the 2009 pH1N1, like that of most swine H1N1 IAV, has only 219 amino acids [60,61,62]. The IAV NS1 protein contains at least four distinct domains: (i) The N-terminal domain (the first 73 amino acids) contains an RNA-binding domain (RBD) [60] (Figure 1B), which forms three α-helices important for the dimerization of the protein and for the binding to dsRNA [63,64,65]. It was shown that residues at positions 38 and 41 of the NS1 RBD affect the binding to dsRNA without affecting NS1 dimerization [65,66]. A nuclear localization signal (NLS) (amino acids 35–41) overlaps with the RBD sequence [67,68]. In addition, most IAV strains encode a second NLS at the C-terminal end (amino acids 216–221) of the protein [67]. (ii) The RBD is followed by a 10–15 amino acids linker (L) domain (Figure 1B), which has high flexibility and connects the RBD and effector domain (ED). (iii) The 88–202 ED (Figure 1B), which contains a β-sheet structure and a long central helix. The ED of most IAV strains contains a nuclear export signal (NES, amino acids 137–146) that favors NS1 protein localization at both the nucleus and the cytoplasm [69]. Notably, more than 50 host proteins have been reported to interact with the NS1 ED (Table 1 and data not shown, reviewed in [70]), although some of these interactions do not occur with all of the NS1 proteins encoded by different IAV strains, and it has been suggested that host-adaptation processes can be responsible for this variability [60,62,71,72,73,74]. Those host proteins include, among others, the 30 kDa subunit of cleavage and polyadenylation specificity factor (CPSF30), the poly(A)-binding protein II (PABPII), the eukaryotic initiation factor 4G (eIF4GI), the poly(A)-binding protein I (PABPI), and multiple host factors involved in antiviral response, such as RIG-I, E3 ligase tripartite motif-containing protein 25 (TRIM25), PKR, or the regulatory subunit p85-β of phosphatidylinositol 3-kinase (PI3K) (reviewed in [70], Table 1). Finally, (iv) the last domain contains a C-terminal tail (CTT) of 11–33 amino acids, which is intrinsically disordered, and includes a PDZ-binding motif, which affects virus pathogenesis and is not present in all IAV NS1 proteins [75,76] (Figure 1B).

### 2.2. Mechanisms of IAV NS1 Protein Inhibition of Innate Immune Responses

**Figure 2:**
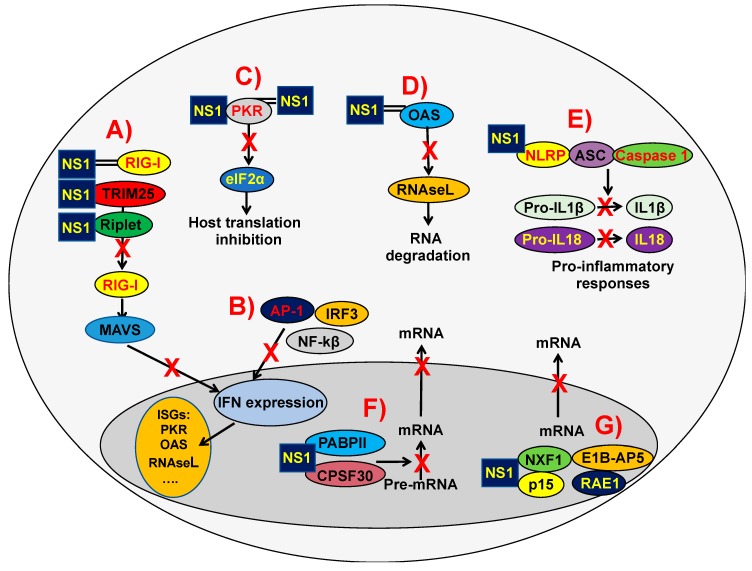
Direct and indirect effects of the IAV NS1 protein on innate immune responses. (**A**) IAV NS1 decreases RIG-I activation, and IFN responses, through the sequestration of dsRNA [88,89,90], or by interaction with TRIM25 or Riplet, which results in the suppressed ubiquitination and activation of RIG-I, which signals through the mitochondrial antiviral signaling protein (MAVS) to induce IFN responses [91,92]. (**B**) NS1 inhibits the IRF3 [99], NF-κβ [117], and AP-1 [100] transcription factors, impairing IFN production, and, therefore, the induction of IFN-stimulated gene (ISG) products. In addition, NS1 directly inhibits the antiviral activities of the ISGs PKR and OAS-RNaseL. (**C**) The IAV NS1 protein binds dsRNA and PKR, leading to decreased PKR activity to phosphorylate eIF2α, and host translation inhibition [101,102,103]. (**D**) The IAV NS1 protein, via the dsRNA-binding activity of its RBD, inhibits OAS activation, blocking RNA degradation [87]. (**E**) The IAV NS1 protein also inhibits NLRP3 inflammasome activation [105,106,107], impairing the cleavage of pro-interleukin (IL)-1β and pro-IL-18 into their mature forms IL-1β and IL-18, respectively, which are released from the cell to stimulate inflammatory processes. (**F**) NS1 proteins from some human and avian IAV strains bind to CPSF30, blocking the cleavage of immature mRNAs (pre-mRNAs) and the recruitment of the poly(A) polymerase to add the poly(A) tail [62,72,74,108,109,110,123]. The IAV NS1 protein also binds to the poly(A)-binding protein II (PABPII), inhibiting its ability to stimulate the synthesis of long poly(A) tails [111]. These last two processes lead to host shutoff of protein synthesis [111]. (**G**) Additionally, the NS1 of influenza A/WSN/33 H1N1 (WSN) binds NXF1, p15, RAE1, and E1B-AP5, which interact with both mRNAs and nucleoporins to direct mRNAs through the nuclear pore complex, blocking their function, and likely facilitating host cellular shutoff [116].

The IAV NS1 protein allows the virus to replicate efficiently by suppressing the host innate immune response by a variety of mechanisms (reviewed in [60,70,77]) (Figure 2), including the direct or indirect interaction with host factors (selected examples are shown in Table 1). Accordingly, IAVs lacking or expressing truncated forms of NS1 [78,79,80,81], expressing reduced levels of NS1 [82], or encoding amino acid mutations affecting NS1 functions [72,82,83,84] are severely impaired in cells competent in the production of type I IFN, whereas they show similar levels of replication in type I IFN-deficient cells (e.g., Vero cells) [78]. Thus, IAV encoding NS1 proteins with an altered anti-IFN function lack virulence in IFN-competent animals, but are pathogenic in mice defective in key proteins of the type I IFN pathway.

The different NS1 protein domains are involved in regulating the host innate immune response, and in this review we will summarize some of the most characteristic functions. It has been shown that the NS1 RBD mutations R38A and K41A inhibit the binding of NS1 protein to dsRNA [65], a function critical for counteraction to innate immune responses, leading to virus attenuation [73,85]. By using primary differentiated respiratory epithelial cell cultures infected with a recombinant virus encoding a mutation in the RBD (R38A), it was revealed that the RBD is a critical regulator of both cytokine production and cytokine sensitivity during IAV infection [86]. Furthermore, using the PR8 backbone and the R38A mutant virus, it was shown that a functional RBD is required for IFN suppression and virulence in mice [73]. Moreover, IAV NS1, via its RBD, inhibits OAS activation because it outcompetes OAS for interaction with dsRNA [87] (Figure 2D and Table 1).

The molecular mechanisms affecting NS1’s ability to counteract innate immune responses include inhibiting RIG-I activation by sequestration of this RNA helicase and its activating ligand [88,89,90], or by interaction with TRIM25 or Riplet, which results in the suppressed ubiquitination and activation of RIG-I [91,92,93] (Figure 2A and Table 1). Notably, IAV encoding NS1 proteins carrying amino acid changes E96A/E97A in a putative protein–protein interaction motif in the ED domain are unable to bind TRIM25, causing virus attenuation and higher IFN induction [91]. However, these amino acid changes are not involved in the inhibition of RIG-I activation mediated by Riplet [92]. Additionally, NS1 indirectly regulates RIG-I signaling by increasing the expression of the ubiquitin-editing protein A20 after infection (Table 1). A20 suppresses IRF3-mediated induction of type I IFN and ISGs [94,95].

NS1 blocks the inhibitor of kappa β kinase (IKK) subunit beta (IKK-β), inhibiting the activation of the NF-κB pathway, and preventing the expression of antiviral genes [98,117] (Figure 2B). Furthermore, NS1 impairs the IKK-mediated phosphorylation of histone 3 (H3) in the nucleus, suppressing the antiviral response [98]. In addition, NS1 inhibits the IRF3 transcription factor [99], and the Jun N-terminal kinase (JNK), a kinase which phosphorylates and thereby increases the activity of transcription factors of the AP-1 family [100] (Figure 2B and Table 1).

The IAV NS1 protein directly inhibits specific ISG products, such as PKR (Figure 2C) and ribonuclease L (RNase L) (Figure 2D) (reviewed in [70]) (Table 1). NS1 inhibits PKR activation by binding throughout NS1 amino acids 123–127 [101,102] and 35 and 46 [103] (Figure 2C, Table 1). Intriguingly, IAV NS1 mutants at residues 35 and 46 were not pathogenic in PKR^+/+^ mice but replicated to high titers in lungs of PKR^−/−^ mice and were lethal, highlighting the antiviral activity of PKR [103].

IAV NS1 also inhibits NLRP3 inflammasome activation [105,106,107] (Figure 2E and Table 1). The NS1 RBD (basic amino acid residues R38 and K41) and TRIM25-binding residues (acidic residues E96 and E97) were required for the suppression of NLRP3-inflammasome-mediated IL-1β secretion [107]. It has been shown that the IAV NS1 protein interacts with NLRP3 [107] and impairs ASC speck formation and suppresses ASC ubiquitination, processes relevant to inflammasome activation [106] (Figure 2E). Moreover, two target lysine residues, K110 and K140, which are essential for both porcine ASC ubiquitination and NLRP3-inflammasome-mediated IL-1β production, were identified [106]. According to these results, it was previously shown that viral mutants either lacking or possessing non-functional RNA-binding and dimerization domains induced 10–50 times more biologically active IL-1β and 5 times more biologically active IL-18 than the wild-type (WT) influenza A/Puerto Rico/8/34 (PR8) H1N1, correlating with an enhanced activity of caspase 1 [118] (Figure 2E).

Another important target of NS1 is the phosphatidylinositol-3-kinase (PI3K) signaling pathway. NS1 activates the PI3K pathway by direct interaction with the p85β subunit [114], causing the phosphorylation of a downstream mediator of PI3K signal transduction, Akt. The NS1–PI3K interaction increases the rate of viral internalization, inhibits apoptosis [119], and enhances type I IFN and pro-inflammatory production by enhancing the activity of IRF3 [120,121]. NS1–p85β interaction is dependent on Y89/M93 [114], L141/E142 [115], and P164/P167 [122] of the effector domain, all of which are located adjacent to each other within a cleft between the two NS1 monomers.

### 2.3. Effect of the IAV NS1 Protein on Inhibition of Gene Expression

NS1 proteins from some human and avian IAV strains bind to CPSF30, blocking the processing of cellular mRNAs [62,72,74,108,109,110,124] (Figure 2F and Table 1). The region of IAV NS1 that binds CPSF30 is largely hydrophobic, and is primarily defined by the amino acid residues K110, I117, I119, Q121, V180, G183, G184, and W187 [108], with W187 being a hydrophobic amino acid that is essential for dimerization of the NS1 ED [108]. The NS1 binding to CPSF30 inhibits the recognition by the CPSF complex of polyadenylation signals at the 3’ end of mRNAs during transcription, blocking the cleavage of immature mRNAs (pre-mRNAs) and the recruitment of the poly(A) polymerase to add the poly(A) tail [123,124]. The poly(A) tail of eukaryotic mRNAs is required for RNA stability, nucleus export, and translation [123]. As a consequence, unprocessed cellular pre-mRNAs accumulate in the nucleus (Figure 2F), leading to an inhibition of general gene expression, including IFN, ISGs encoding antiviral activities, and pro-inflammatory genes [70].

The IAV NS1 protein also binds to the poly(A)-binding protein II (PABPII) (Figure 2F and Table 1), inhibiting the ability of PABPII to stimulate the synthesis of long poly(A) tails [111]. Because of this interaction, cellular pre-mRNAs that contain short poly(A) tails (around 12 nucleotides) accumulate in the nucleus of IAV-infected cells [111] (Figure 2F). The binding of the IAV NS1 protein to CPSF30 and PABPII block pre-mRNA processing and the nuclear export of mRNAs, therefore leading to a general inhibition of host gene expression [70,110,111].

The IAV NS1 protein also enhances the translational rate of viral, but not cellular, mRNAs [113]. Using A/Victoria/3/75 H3N2, it was shown that NS1 binds the eukaryotic initiation factor 4GI (eIF4GI) (Table 1), the large subunit of the cap-binding complex eIF4F, suggesting that NS1 recruits eIF4GI specifically to the 5’ untranslated region (5’ UTR) of the viral mRNA, allowing for the preferential translation of the IAV mRNAs, which likely leads, indirectly, to host cellular shutoff [113].

The NS1 protein of the classical swine H1N1 IAV encoded 230 amino acids until the mid-1960s, when a stop codon emerged at position 220 and caused an 11 amino acid truncation at the C-terminus [127]. This 219 amino acid NS1 has subsequently been predominant in the classical swine H1N1, and emerged in the 2009 pH1N1 IAV [112]. The C-terminal-truncated NS1 of the 2009 pH1N1 virus was inefficient at blocking host gene expression, while an extension of the truncated NS1 to its full-length 230 amino acid form increased the ability of the protein to inhibit host gene expression [112]. The mechanism that mediates the increased inhibition of host gene expression by the full-length NS1 involved the restoration of NS1’s binding to PABPII [112]. In vitro and in vivo characterization of two 2009 pH1N1 viruses encoding the C-terminal 11 amino acid truncated 220 amino acids and the full-length 230 amino acid NS1 showed that the C-terminal 11 amino acid truncation did not significantly affect virus replication. However, this truncation increased virus pathogenicity in mice by causing more severe hemorrhage and alveolitis in the lungs [112].

The H3N2 IAV circulating in humans from ∼1950 to ∼1987 encoded an NS1 protein with a 7 amino acids C-terminal extension (237 amino acids in total). No significant differences in the ability to counteract IFN responses were observed between proteins encoding the NS1 230 and 237 amino acid proteins [61]. However, the virus encoding the 237 amino-acid-length NS1 protein outcompeted the mutant virus during mixed infections, suggesting that the 7 amino acid extension in NS1 slightly affected the growth or fitness of the virus [61].

Recently, it was found that the NS1 protein can bind cellular dsDNA, preventing the loading of the transcriptional machinery to the DNA, decreasing the expression of antiviral genes [128]. The residues R38 and K41 in the NS1 RBD are important to this binding [128].

Additionally, the NS1 of the IAV WSN binds many components of the mRNA’s export machinery: nuclear RNA export Factor 1 (NXF1), p15, Ribonucleic Acid Export 1 (RAE1), and adenovirus early region 1B-associated protein 5 (E1B-AP5), which interact with both mRNAs and nucleoporins to direct mRNAs through the nuclear pore complex, blocking their function [116] (Figure 2G and Table 1). These data indicate different cellular mechanisms mediating the inhibition or regulation of host gene expression by the IAV NS1 protein.

### 2.4. Effect of IAV NS1 on Virus Replication, Induction of Cytokines, and Pathogenesis

#### 2.4.1. Effect of Partial and Complete NS1 Deletions on Virus Replication, Induction of Cytokines, and Pathogenesis

Viruses encoding truncated NS1 proteins are severely impaired in replication in cultured cells, and are highly attenuated in vivo, because of defects of the truncated NS1 proteins to counteract IFN responses. Examples include a PR8 H1N1 virus encoding the first 99 or 126 amino acids of NS1 [81,129,130], H5N1 viruses encoding the first 73, 99, and 126 amino acids of NS1 [131], an A/Texas/36/91 H1N1 virus encoding the first 126 amino acids of NS1 [132], and equine Influenza virus (EIV) [133,134] and canine Influenza virus (CIV) [80], which are H3N8 viruses encoding the first 73, 99, and 126 amino acids of NS1.

Infection with a PR8 H1N1 virus encoding an NS1 protein expressing only the first 125 amino acids (PR8 NS1–125) induced significantly higher amounts of IFN-β, IL-6, C-C motif Chemokine ligand 3 (CCL3), macrophage inflammatory protein 1-alpha (MIP-1α), and tumor necrosis factor (TNF) than the parental PR8 virus. Nevertheless, the PR8 NS1–125 virus was as efficient as the parental PR8 virus in inhibiting IL-1β and IL-18 release in infected macrophages [118]. Mutant viruses lacking or possessing nonfunctional RBD and dimerization domains induced significantly higher amounts of biologically active IL-1β and IL-18 than the parental or PR8 NS1–125 viruses [118]. In infected macrophages, these defective viruses induced rapid apoptosis, correlating with the enhanced activity of caspase-1. These results indicated that the N-terminal domain of the PR8 NS1 protein might control caspase-1 activation, leading to a repression in the maturation of pro-IL1β-, pro-IL18-, and caspase-1-dependent apoptosis in infected human macrophages [118].

#### 2.4.2. Effect of NS1 Mutations Affecting NS1-Mediated Host Shutoff on Innate Immune Responses and Virus Pathogenesis

IAV encoding amino acid changes critical for NS1-mediated host shutoff showed altered pathogenesis. For example, a single amino acid change (I106M) in the NS1 protein from an avian-derived H7N9 IAV (A/Shanghai/1/2013 and A/Shanghai/2/2013 strains) restores the NS1’s ability to bind CPSF30 and the ability to block host gene expression, including IFN response genes [135]. Furthermore, a recombinant H7N9 virus expressing NS1 I106M replicated to higher titers in vivo and is subtly more virulent than the parental virus [135] (Table 2). Notably, PR8 NS1 cannot bind CPSF30 due to mutations at positions 103 and 106 [73]. Interestingly, the restoration of CPSF30 binding by introducing amino acid changes at these amino acid residues (L103F and I106M) resulted in enhanced virulence [73]. Likewise, using the highly pathogenic A/Hong Kong/483/97 H5N1 virus, it has been shown that the amino acid changes L103F and I106M increase the binding of NS1 protein to CPSF30, the ability to inhibit host gene expression, and virus pathogenicity in mice [74,136] (Table 2). The increased virulence of the H5N1 virus is associated with a faster systemic spread of the virus, particularly to the brain, where increased viral replication and severe pathology occur [136]. This augmented spread was also associated with increased cytokine and chemokine levels in extrapulmonary tissues [136].

In general, the NS1 proteins of the H3N2 and H2N2 IAV bind CPSF30, inhibiting general host gene expression [137]. However, we have recently shown that an amino acid change I64T in the NS1 protein of a virus from a patient infected with a seasonal H3N2 IAV leads to an impairment in its ability to bind CPSF30, and, therefore, a lack of inhibition of host gene expression [83] (Table 2). Furthermore, this mutation also led to reduced virus replication in cultured cells and to virus attenuation in vivo [83]. Similarly, viruses encoding the D189N and V194I amino acid changes in seasonal H3N2 IAV lead to NS1 proteins showing decreased binding to CPSF30, and reduced NS1-mediated inhibition of host gene expression [84] (Table 2). Similar to the virus encoding the mutation I64T, IAV containing the D189N and V194I amino acid changes were attenuated in vivo, with the virus containing the V194I amino acid change more attenuated than the virus with D189N, most likely because the V194I substitution confers a temperature-sensitive phenotype [84].

**Figure 3:**
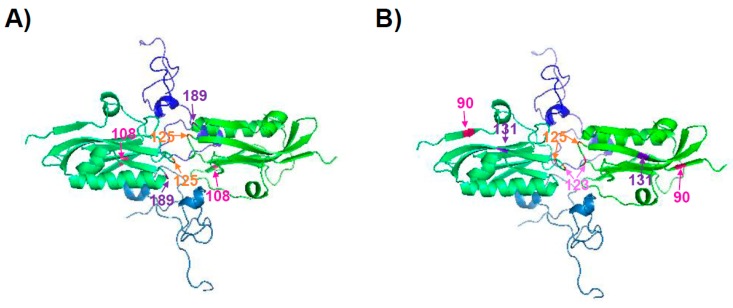
The tridimensional structure of the IAV NS1 ED coupled to the F2/F3 domain of CPSF30. A/Udorn/72 H3N2 strain NS1 ED bound to the F2/F3 fragment of the human CPSF30 was previously crystalized [108] (protein data bank (PDB) entry 2RHK). Colors were included using the MacPyMOL Molecular Graphics system (pymol.org). Each monomer of the NS1 ED is represented in green colors. Monomers of the F2/F3 fragment of the human CPSF30 are represented in blue colors. The artificially introduced NS1 amino acid residues 108, 125, and 189 restoring NS1–CPSF0 binding (**A**) [125], and the residues 90, 123, 125, and 131 found in naturally circulating pH1N1 viruses (**B**) [126], are indicated in reddish colors (orange, purple, and pink). Figure adapted from [126].

The NS1 protein encoded by pH1N1 viruses at the beginning of the 2009 pandemic did not inhibit general gene expression because of a lack of interaction with CPSF30 [125]. Interestingly, by introducing three amino acid changes (R108K, E125D, and G189D), the pH1N1 NS1 protein regained the ability to interact with CPSF30 (Figure 3A), and was more efficient at antagonizing host innate immune responses in primary human epithelial cells than its WT counterpart [125] (Table 2). However, the amino acid changes R108K, E125D, and G189D had no significant effect on virus replication in human or swine tissue culture cells [125]. Surprisingly, in a mouse model of infection, the pH1N1 mutant virus containing the NS1 R108K, E125D, and G189D amino acid changes appeared to cause less morbidity and was cleared faster than the original 2009 pH1N1 virus [125]. The mutant virus also grew with reduced titers in the upper respiratory tracts of ferrets [125]. We have recently described that currently circulating pH1N1 viruses encode an NS1 with six amino acid changes (E55K, L90I, I123V, E125D, K131E, and N205S) compared to the viruses circulating at the origin of the 2009 pandemic [126]. Remarkably, these six amino acid changes increased the ability of the pH1N1 NS1 protein (pH1N1/NSs-6mut) to inhibit host gene expression, mainly by restoring the binding of NS1 to CPSF30 [126] (Table 2). Some of these residues (i.e., 90 and 131) are far from the NS1–CPSF30 interface (Figure 3B). However, they still could be affecting the NS1 binding to CPSF30 by affecting the conformational structure of residues near or in the protein–protein interface. Moreover, they could be affecting the formation of NS1 dimers. As a consequence, a recombinant pH1N1 virus containing these six residue changes in the NS1 protein (pH1N1/NSs-6mut) inhibited innate IFN and proinflammatory responses more efficiently than the parental pH1N1 virus. However, virus titers were similar in cell cultures and in mouse lungs, and the virus containing the six amino acid changes (pH1N1/NSs-6mut) was partially attenuated as compared to the pH1N1 WT [126]. Notably, the pH1N1/NSs-6mut virus induced decreased levels of pro-inflammatory cytokines, likely due to the increased inhibition of host gene expression mediated by interaction of the evolved NS1 protein with CPSF30 [126]. These diminished levels of inflammation induced by the pH1N1/NSs-6mut virus are likely responsible for the attenuated disease phenotype, most likely representing a mechanism of host–virus adaptation affecting viral pathogenesis [126]. Moreover, these data suggest a selective advantage for viruses encoding an NS1 protein that is able to inhibit general gene expression in the human host, as viruses encoding NS1 proteins that are able to inhibit general host gene expression are selected in the population.

Moreover, it has been shown that a K186E amino acid substitution in the H3N8 CIV (A/canine/NY/dog23/2009 strain) and EIV (A/equine/Ohio/1/2003 strain) IAV restores their ability to bind CPSF30 and inhibit host gene expression [62,72] (Table 2).

#### 2.4.3. Effect of NS1 Mutations Affecting Inflammatory Processes

A G45R amino acid change in the NS1 protein of PR8 H1N1 contributes to virulence by increasing the expression of pro-inflammatory cytokines in mice [139]. PR8 viruses encoding the NS1 gene from a high pathogenic avian A/Hong Kong/156/97 H5N1 virus were resistant to IFN and TNF antiviral activities and showed increased pathogenicity in pigs [140] and in mice [141]. Interestingly, a glutamic acid (E) at position 92 in the A/Hong Kong/156/97 H5N1 NS1 protein was critical for this phenotype, as an E92D mutant virus grew with lower titers and was attenuated in pigs [140] and in mice [141]. Mice infected with a virus encoding the NS1 protein of A/Hong Kong/156/97 H5N1 exhibited elevated pulmonary concentrations of the inflammatory cytokines IL-1α, IL-1β, IL-6, and IFN-γ and the chemokine CXCL10, and decreased concentrations of the anti-inflammatory cytokine IL-10 [141]. This cytokine imbalance is likely responsible for the unusual severity of the IAV H5N1 and correlates with the clinical findings observed in two humans who died of IAV H5N1 infection [141].

It is becoming clearer that the morbidity and pathogenesis caused by IAV are consequences of the inflammatory response [142]. Therefore, the inhibition of inflammasome activation by the IAV NS1 protein likely contributes to decreasing virus virulence [105,106,107]. However, more studies are needed to analyze the relevance of NS1-mediated inflammasome inhibition in virus pathogenesis, since most of the studies were performed in cell culture and not in animal systems. Therefore, the effect of NS1 mutations on IFN and pro-inflammatory cytokine induction likely has positive or negative effects of virus replication and pathogenesis, depending on the balance between IFN and pro-inflammatory responses. In addition, we and others have shown that other viral proteins could have a role in the modulation of the host innate immune system, and the interplay between NS1 and other viral factors (e.g., PA-X), which is discussed later in this review, will require more investigation.

### 2.5. Modulation of IAV NS1 Expression for the Development of More Efficient Live-Attenuated Influenza Vaccines (LAIV)

IAV infections represent a severe public health and economic concern that is most effectively prevented through vaccination [143]. However, despite the application of effective and broad vaccination programs, IAV infections are responsible for global epidemics every year, and the efficiency of current influenza vaccines is suboptimal [144,145]. The decreased efficacy of seasonal influenza vaccines is in part due to an antigenic drift in the two main proteins (HA and NA) responsible for inducing neutralizing antibodies [12,145,146,147,148]. Therefore, there is an urgent need to develop more effective vaccines against influenza viruses. Currently, three types of influenza virus vaccines have been approved for their use in humans by the U.S. Food and Drug Administration (FDA): inactivated influenza vaccines (IIV), live-attenuated influenza vaccines (LAIV), and recombinant viral HA [143,145,149,150]. IIV is the most common vaccine for the control of influenza infections, and it has been shown to elicit protective humoral immunity [144,145]. However, it is known that LAIV elicit more rapid and broader efficient innate and adaptive immune responses than IIV, and can provide more efficient cross-reactive T-cell-mediated protection against heterologous influenza viruses [143]. Commercially available LAIV are based on the cold-adapted, temperature-sensitive, attenuated master donor virus (MDV) A/Ann Arbor/6/60 H2N2 that replicates in the upper respiratory tract but does not damage the lower respiratory tract due to the elevated temperatures that restrict viral replication [151]. However, new approaches to attenuate the virus(es) in the vaccine are also needed to overcome the limitations that are associated with the current LAIV [143,145,148].

IAV lacking the NS1 gene or encoding truncations in the NS1 gene are attenuated in vivo. Therefore, they represent promising LAIV candidates for the prevention of influenza infections [81]. For example, a PR8 virus expressing only the first 126 amino acids of NS1 was attenuated in mice and fully protects against a lethal viral challenge, even in aged mice [129]. Similarly, viruses expressing only the first 73 or 126 amino acids of the NS1 protein of A/NewYork/1682/2009 pH1N1 protected mice and ferrets from WT viral challenge [152]. Vaccination with a virus expressing only the first N- terminal 126 amino acids of A/Texas/36/1991 H1N1 was also effective in protecting macaques from homologous WT viral challenge [132]. This protection was in the absence of significant or long-lasting adverse effects and through the induction of a robust adaptive immune response [132]. Likewise, A/canine/NY/dog23/2009 H3N8 viruses encoding truncated (NS1–73, NS1–99, and NS1–126) or lacking (ΔNS1) NS1 were reduced in their ability to replicate ex vivo and in vivo [80]. Moreover, immunization of mice by a single intranasal dose of these NS1-deficient or truncated mutant viruses protected against WT H3N8 CIV challenge [80]. Similarly, an equine H3N8 virus (A/equine/Kentucky/5/2002) encoding a truncated NS1 (NS1–126) was attenuated in vivo and provided protection against WT EIV challenge in horses [133,134]. Remarkably, a clinical trial in phase I/II in humans showed that a replication-deficient trivalent influenza vaccine containing the IAV strains A/Brisbane/59/07 H1N1-like, A/Brisbane/10/07 H3N2-like, and B/Florida/04/06-like, all lacking the NS1 protein, is safe and induces significant levels of antibodies that protect against disease caused by the influenza virus [153].

We have explored another strategy for generating LAIV based on NS1 modifications [82]. We have been able to generate recombinant influenza PR8 viruses encoding misrepresented mammalian codons (codon deoptimization, CD) comprising the entire *NS* gene (NS1 and NEP) or the mRNA corresponding to the individual viral NS1 or NEP, without modifying the respective splicing and packaging signals [82]. The growth of these synthetic CD PR8 viruses was impaired in vivo, while they retained immunogenicity, and conferred protection upon a single intranasal administration dose, against both homologous (PR8, H1N1) and heterologous (X31, a reassortant virus carrying HA and NA proteins from an H3N2 virus) viral challenges [82]. These results open the possibility of using this NS CD approach, alone or in combination with other attenuation strategies, for their implementation as LAIV.

Recently, a systematic approach for vaccine development that eliminates IFN-modulating functions while maintaining virus replication fitness has been described [154]. Using this approach, a highly IFN-sensitive virus, encoding three amino acid changes in PB2, three amino acid changes in M1, and two amino acid changes in NS1, was highly attenuated in IFN-competent hosts. However, this virus was able to induce transient IFN responses, and robust cellular and humoral responses, providing protection against homologous and heterologous challenges [154].

## 3. The IAV PA-X Protein

**Figure 4:**
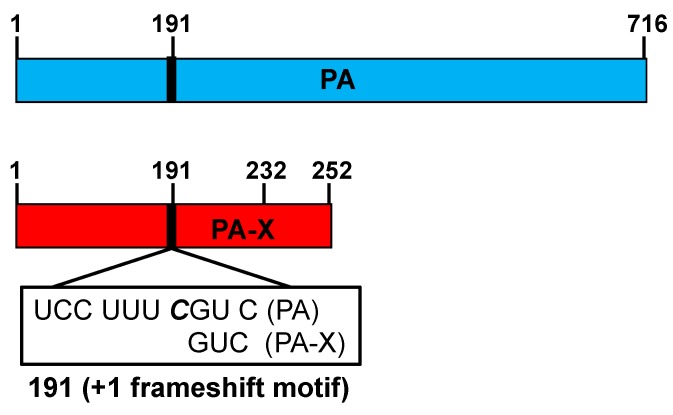
A schematic representation of the IAV PA viral segment and the PA and PA-X open reading frames (ORFs). Blue and red boxes indicate the ORF for PA and PA-X, respectively. The +1 frameshift motif (UCC UUU ***C***GU C) at position 191 is indicated. Bold and italics in the frameshift motif (C nucleotide) indicate that the nucleotide C is not read during PA-X translation. PA-X proteins containing 232 or 252 amino acids if the C-terminal region has a 41 or 61 amino acid extension, respectively, are indicated.

IAV segment 3 encodes the PA and the PA-X proteins [155]. PA is translated directly from the PA mRNA, and is required for virus replication and transcription, together with the other components of the viral RNA-dependent RNA polymerase (RdRp), PB2 and PB1 [156]. PA-X is translated as a +1 frameshift open reading frame (ORF) from the PA viral segment [155] (Figure 4). During translation, the ribosome shifts at a specific sequence in the PA mRNA, a U-rich region followed by a rare codon [157]. These rare codons promote ribosomal frame-shifting because they are typically decoded more slowly [157]. Because of the +1 frameshift, during the translation of PA, the codons C**GU C**AG (amino acids RQ) are read as **GUC** (V), not reading the C nucleotide during PA-X translation and producing a shift in the open reading frame (Figure 4) [155]. PA-X shares the same first N-terminal 191 amino acids with the PA protein, including the endonuclease domain (Figure 4). However, the ribosomal frameshift produces a PA-X containing a unique short C-terminal sequence [155]. Most of the human IAV strains contain a 61 amino acids C-terminal extension (252 amino acid PA-X), with the important exception of the 2009 pH1N1 viruses that encode a 41 amino acids extension (232 amino acid PA-X) [158] (Figure 4).

### 3.1. The IAV PA-X Protein’s Effect on Host Gene Expression

PA-X induces the shutoff of host protein expression in infected cells, contributing to the blocking of cellular antiviral responses [124,155,159,160,161,162,163]. However, PA-X-induced suppression of host protein synthesis not only affects antiviral genes, and the effect of PA-X expression on virus fitness and virulence seems to be strain-specific [158,162,163,164,165,166]. The PA-X-mediated shutoff activity involves the N-terminal endonucleolytic domain, resulting in the degradation of host mRNAs. Accordingly, mutations in the endonuclease active site of PA-X inactivate the protein’s ability to induce host cellular shutoff [167]. PA-X selectively targets cellular mRNAs while not affecting viral mRNAs, thereby ensuring successful viral replication and counteracting the antiviral response in the host [160]. PA-X selectively degrades host RNA polymerase II (Pol II)-transcribed mRNAs and non-coding RNAs in the nucleus of infected cells, while sparing the products of polymerases I and III [124,161]. Complete degradation of host mRNAs following PA-X-mediated endonucleolytic cleavage is dependent on the host 5’->3’-exonuclease Xrn1 [161]. The host protein shutoff activity of IAV PA-X is stronger than that of PA or the N-terminal PA domain, indicating that the C-unique terminal region of PA-X increases inhibition of host protein expression [159,168]. Two independent studies demonstrated that a PA-X expressing the first 15 amino acids in the C-terminal region (positions 192–206) displays the same host cellular shutoff activity as the full-length protein, and that six basic amino acids (195R, 198K, 199R, 202K, 203K, and 206K) play a key role in PA-X’s ability to inhibit host gene expression [167,169]. In addition, other studies reported that amino acids 233–252 in the C-terminal region (found in most IAV strains, but not in the 2009 pH1N1 IAV strains) of PA-X enhance the suppression of host gene expression of human (pH1N1) or avian (H5N1 and H9N2) IAV strains [166,170].

Cellular host protein shutoff activity is strongly associated with nuclear accumulation of the PA-X protein, which is mainly mediated by four conserved basic residues (198K, 199R, 202K, and 203K) in the C-terminal region of PA-X [161]. Supporting these results, a recent study found that the first nine amino acids in the C-terminal region of PA-X (amino acids 192–200) are sufficient for nuclear localization, and that three basic amino acids (195K, 198K, and 199R) are key for host shutoff ability and PA-X’s nuclear accumulation [167]. Accordingly, an additional study revealed that PA-X mutants with amino acid changes at positions 4, 9, 24, 27, 39, 45, 87, 94, 106, 107, 108, 119, 120, 123, 124, 125, 148, 154, 160, 163, 168, and 171 predominantly localized in the cytoplasm and displayed decreased shutoff activity, suggesting that these mutations decreased the shutoff activity of PA-X by affecting PA-X´s translocation to the nucleus [171]. Other studies indicate that PA-X degrades mature mRNAs localized both in the nucleus and in the cytoplasm. However, degradation of mRNAs by PA-X in the nucleus was more efficient than in the cytoplasm, thereby showing that PA-X’s ability to degrade host cellular mRNAs occurs at different functional sites [167].

### 3.2. Effect of IAV PA-X on Virus Pathogenesis and Suppression of Innate Immune Responses

The strain-specific loss of PA-X expression can increase or decrease viral replication and virulence. For example, loss of PA-X expression in the 2009 pH1N1 A/Beijing/16/2009 strain and in the highly pathogenic avian A/Anhui/1/2005 and A/chicken/Jiangsu/K0402/2010 H5N1 strains increased viral replication, virulence, and host inflammatory response in mice [172,173] and in birds (A/chicken/Jiangsu/K0402/2010 strain) [163]. Similarly, PA-X expression was found to decrease the pathogenicity of the pandemic A/Brevig Mission/1/1918 H1N1 virus in mice [155]. Recently, we have found that the currently circulating pH1N1 viruses have evolved to encode four amino acid changes in the PA-X protein (V100I, N204S, R221Q, and L229S) [71]. These amino acid differences between early (2009) and more recent (2015) pH1N1 isolates are responsible for decreased PA-X-mediated shutoff, including innate immune response genes [71]. Higher pro-inflammatory responses that correlate with increased virulence were observed after infection with recombinant viruses expressing the PA-X protein from currently circulating viruses that have a decreased host shutoff activity [71]. In contrast, recent work has described that loss of PA-X in the A/California/04/2009 pH1N1 or A/chicken/Hebei/LC/2008 H9N2 viruses leads to a reduction in viral replication and pathogenicity as well as an increased host innate immune response in mice [159,165,166]. These studies suggest that the effect of PA-X on virus replication and pathogenesis could be host- and strain-specific, perhaps reflecting adaptation to a given host.

The biological significance of PA-X’s length was studied by generating human A/Beijing/16/2009 and A/California/04/2009 pH1N1, and the avian A/tree sparrow/Jiangsu/1/2008 H5N1 and A/chicken/Hebei/LC/2008 H9N2 recombinant viruses, encoding 61 (192–252) or 41 (192–232) amino acids at the C- terminal region [166,170]. The results from two independent studies suggested that the last 233–252 amino acid extension slightly increased viral replication and virulence in mice, strengthened the viral-induced inflammatory response and apoptosis, and elevated the host shutoff ability by PA-X as compared to those encoding the 41 amino acid extension [166,170].

### 3.3. Modulation of PA-X to Generate LAIVs

It has been described that inhibition of PA-X expression leads to a slight virus attenuation of certain IAV strains [165,166]. Therefore, this strategy could be used in some IAV strains as an additional measure to generate LAIV. Interestingly, mice infected with a A/California/04/2009 pH1N1 expressing lower levels of the PA-X protein induced higher titers of IFN expression and grew with lower titers in the lungs than the parental pH1N1 virus [159]. However, slightly higher titers of anti-HA and neutralizing antibodies were likely produced after infection with the PA-X mutant pH1N1 virus [159] (Table 3), a critical aspect for the induction of protective immune responses, which should be further analyzed.

## 4. Interplay of the IAV NS1 and PA-X Proteins

### 4.1. Interaction of the IAV NS1 and PA-X Proteins on Virus Fitness and Virulence

The IAV NS1 and PA-X proteins have the synergistic ability to inhibit host gene expression, although using different mechanisms. However, whereas many studies have described the individual effect of IAV NS1 and PA-X on host gene expression, the interplay between these two proteins has been barely studied. We have described that recombinant LAIV pH1N1 viruses encoding NS1 and PA-X proteins that simultaneously inhibit or do not inhibit host gene expression are impaired in viral growth properties in cultured cells, correlating with virus attenuation in vivo, in comparison to recombinant pH1N1 viruses in which only one of the viral proteins (NS1 or PA-X) inhibited host gene expression [174] (Table 3). Remarkably, by using a pH1N1 LAIV as a backbone, we have observed greater differences in virus pathogenicity than those detected using a WT virus, suggesting that the LAIV virus could be a better option to study virulence factors and virus–host interactions. Importantly, contrary to other studies [166], we did not observe a higher innate or humoral response with the PA-X-deficient virus in mice, which is most likely due to the reduced viral replication of the attenuated virus [174]. These results suggested, for the first time, that inhibition of host protein expression by IAV is subject to a strict balance that can determine the successful progression of viral infection [160,174]. This knowledge raises questions about the interpretation of experiments that only include mutations in one but not both viral proteins and further demonstrates how virulence in IAV is a multigenic factor where more than one viral gene should be considered.

Multiple mutations have appeared, and they are most likely associated with host adaptation [2,15,125,126]. As indicated above, the NS1 protein of the currently circulating pH1N1 IAV has gained the ability to inhibit host gene expression by acquiring the amino acid substitutions E55K, L90I, I123V, E125D, K131E, and N205S [126]. Based on our previous observation with the LAIV pH1N1 [174], we assessed the ability of PA-X from recent pH1N1 IAV strains with mutated NS1 proteins to inhibit host gene expression [71]. Interestingly, we observed multiple amino acid changes in the PA-X protein (V100I, N204S, R221Q, and L229S). While amino acid changes led to increased NS1-mediated inhibition of host gene expression, changes in recent pH1N1 strains resulted in decreased PA-X-mediated inhibition of host gene expression [71]. Importantly, a recombinant pH1N1 virus containing PA, PA-X, and NS1 genes from currently circulating viruses replicated to higher titers in culture cells and in mice, was more virulent in vivo, and induced a higher activation of pro-inflammatory responses in the lungs [71] (Table 3).

Importantly, these results suggest that a balance in the ability of the NS1 and PA-X proteins to regulate host cellular shutoff is beneficial for IAV [71], confirming our previous findings using the pH1N1 LAIV [174] (Table 3). Although a strong inhibition of host gene expression, including genes with antiviral activity, during viral infection is likely beneficial for IAV, an excessive inhibition of host protein synthesis could affect the expression of multiple host factors required for efficient virus replication, which could be deleterious for the virus [160]. On the other hand, a virus without the ability to block inhibition of host gene expression, including host genes involved in antiviral defense, could be severely attenuated [160]. Thus, a balance in inhibition of host gene expression mediated by IAV NS1 and PA-X proteins is important for viral fitness not only in cultured cells but also in vivo [71,174]. More research into how NS1 and PA-X activities are balanced in different IAV strains and the effects of this balance on viral replication and pathogenesis are really needed. Moreover, other viral factors, such as PB1-F2 [37] or PB2 [175], could also play a key role in modulating the antiviral response induced during IAV infection. These results also demonstrate the need to monitor IAV for co-evolution in the viral genome that could result in more pathogenic viruses or potential pandemics threats.

### 4.2. Inhibition of Host Gene Expression by IAV NS1 and PA-X Proteins for the Development of More Efficient LAIV

We have hypothesized that modulating the ability of NS1 and/or PA-X to counteract the innate immune responses could be a suitable strategy to generate improved LAIV [174]. Therefore, we have explored how the interplay between PA-X and NS1 affects the development of attenuated IAV strains that might be used as vaccines [143]. We have tested a set of A/California/04/09 pH1N1 LAIV encoding PA-X and NS1 proteins with different abilities to inhibit host gene expression [174]. Antibodies specific to total viral proteins, HA, and NA were detected at similar levels in sera from mice infected with viruses where only one of the proteins (PA-X or NS1) has the ability to inhibit gene expression. However, and consistent with the levels of viral replication, antibody levels were lower in serum samples from mice infected with viruses where both proteins have or do not have the ability to inhibit host gene expression [174]. Those data suggest the feasibility of genetically controlling the ability of IAV PA-X and NS1 proteins to inhibit host immune responses, and the feasibility of implementing this approach, alone or in combination with other methodologies, for the development of more effective LAIV to combat disease caused by this important respiratory pathogen [174].

Since the MDV of the IAV LAIV remains constant between seasons, and only the viral HA and NA genes are updated [176], it has been suggested that pre-existing immunity to the internal proteins of the MDV, such as NS1 and PA-X, could limit the response to the LAIV, which must replicate in order to be immunogenic and provide protection against subsequent viral infections. In fact, this could be one of the reasons for the low efficacy of LAIV [13,177]. Understanding how seasonal IAV vaccines are influenced by a pre-existing immunity will be important for developing next-generation IAV vaccines. Moreover, futures strategies to develop new vaccines against IAV, which are highly desired, might require modifying the MDV A/Ann Arbor/6/60 H2N2 currently used for the preparation of seasonal and pandemic LAIV.

## 5. Conclusions

IAV and other viruses hijack the cellular machinery for the progression of viral infection. In order to replicate in the host, IAV encode two viral proteins, NS1 and PA-X, that have developed distinct mechanisms to counteract innate immune responses and the antiviral state produced in the infected and neighboring cells [60,70,160]. Interestingly, one synergistic mechanism used by IAV NS1 and PA-X proteins to counteract the immune response consists in their ability to block host protein synthesis, although using different mechanisms [70,74,155,160,161]. Blocking cellular protein expression contributes to dampening the antiviral response as well as to diverting the cellular machinery towards viral replication [160]. Moreover, controlling the inflammatory response could be important for host survival during viral replication and spread. However, an exacerbated blocking of host protein synthesis can be deleterious and may affect successful viral replication [160]. Likewise, the lack of control of host protein expression would result in the induction of antiviral responses to control viral infection [160]. Therefore, an optimal control of host gene expression and innate immune responses are required for efficient viral replication in IFN-competent systems [160]. It has been reported that IAV PA-X and NS1 proteins are important contributors to maintain this balance, although other viral and host factors could also be playing an important role in this phenomenon. Modulating the ability of IAV to control the antiviral response produced during infection could be a feasible strategy to develop better LAIV [174]. Moreover, surveillance of those virulence factors will be important to predict new pandemics threats. Finally, monitoring the evolution of IAV NS1 and PA-X and their respective ability to inhibit host gene expression will provide important information on the mechanism(s) of viral host adaptation [71].

## Figures and Tables

**Table 1:** Interactions of the IAV NS1 protein with cellular proteins that are involved in innate immune responses.

Host Factor ^a^	NS1 Domain ^b^	Protein Function ^c^	Reference
TRIM25	RBD and ED	Ubiquitin E3 ligase and ISG15 E3 ligase. Modification of RIG-I by poly-ubiquitin chains synthesized by TRIM25 is essential for RIG-I activation	[91,92]
Riplet	RBD and ED	E2-dependent E3 ubiquitin-protein ligase. Modification of RIG-I by poly-ubiquitin chains synthesized by Riplet is essential for RIG-I activation	[92]
RIG-I	RBD	Cytoplasmic PRR of viral nucleic acids, activating innate immune responses	[96]
hPAF1C	CTT	Transcription of Hox and Wnt genes and histone modifications	[97]
IKKβ	CTT	Serine kinase involved in NF-κB activation and IFN and proinflammatory responses	[98]
IRF3	Indirect effect	Transcriptional regulator factor of type I IFN-dependent immune responses	[99]
AP-1	Indirect effect	Transcription factor involved in IFN and pro-inflammatory cytokines induction	[100]
A20	Indirect effect	Ubiquitin-editing enzyme that contains both ubiquitin ligase and deubiquitinase activities. Suppresses IRF3-mediated IFN induction	[94,95]
PKR	RBD and ED	IFN-induced dsRNA-dependent serine/threonine-protein kinase leading to protein translation inhibition	[101,102,103]
RNase L	Indirect effect	Endoribonuclease leading to host gene expression inhibition by mRNA degradation	[87]
PACT	NS1	Activation of EIF2AK2/PKR	[101,104]
OAS	RBD and indirect effect	Polymerizes higher oligomers of 2’-5’-oligoadenylates that bind to RNaseL leading to its activation	[51,87]
NLRP3	ED and indirect effect	Sensor component of the NLRP3 inflammasome, leading to inflammatory responses	[105,106,107]
CPSF30	RBD and ED	Processing of mRNAs, necessary for host gene protein expression	[62,72,74,108,109,110]
PABPII	CTT	Formation of mRNA precursors adding a poly(A) tail, necessary for gene expression	[111,112]
eIF4GI	RBD and ED	Recognition of the mRNA cap, ATP-dependent unwinding of 5’-terminal secondary structure and recruitment of mRNA to the ribosome.	[113]
p85β	ED	PI3K subunit	[114,115]
NXF1	RBD and ED	Nuclear export of mRNA	[116]
RAE1	RBD and ED	mRNA nucleocytoplasmic transport	[116]
P15	ED	Nuclear export of mRNA	[116]
E1B-AP5	RBD and ED	Transcriptional regulator	[116]

^a^ TRIM25: E3 ligase tripartite motif-containing protein 25; RIG-I: pattern recognition receptor (PRR) retinoic acid-inducible gene I; hPAF1C: human PAF1 complex; IKK-β: inhibitor of kappa β kinase; IRF-3: Interferon regulatory factor; PKR: protein kinase R; PACT: protein activator of the interferon-induced PKR; OAS: 2´-5´-oligo A synthetase; NLRP3: NOD-like receptor family member LRR- and Pyrin domain containing-3; CPSF30: 30 kDa subunit of cleavage and polyadenylation specificity factor; PABPII: poly(A)-binding protein II; eIF4G: eukaryotic initiation factor 4G; NXF1: nuclear RNA export Factor 1; RAE1: Ribonucleic Acid Export 1; E1B-AP5: adenovirus early region 1B-associated protein 5. ^b^ RBD: Receptor binding domain; ED: effector domain; CTT: C-terminus tail; ^c^ Protein function description. IFN; interferon.

**Table 2:** The IAV NS1 amino acid residues that are involved in the interaction with CPSF30.

NS1 Amino Acid Residue	IAV	Reference
184	A/Udorn/72 H3N2	[108]
103 and 106	A/Puerto Rico/8/34 H1N1 A/Hong Kong/483/97 H5N1	[74,108,138]
108, 125, 189, 55, 90, 123, 125, 131, and 205	A/California/04/09 H1N1	[125,126]
106	A/Shanghai/1/2013 H7N9 A/Shanghai/2/2013 H7N9	[135]
186	A/canine/NY/dog23/2009 H3N8 A/equine/Ohio/1/2003 H3N8	[62,72]
64, 189, and 194	A/Victoria/361/2011 H3N2 A/Perth/16 H3N2	[83,84]

**Table 3:** Properties of live-attenuated influenza vaccines (LAIV) and currently circulating wild-type (WT) pH1N1 viruses.

Properties	pH1N1 LAIV (ref. [174])				pH1N1 WT (ref. [71])			
	PA_WT_ NS1_WT_	PA_MUT_ NS1_WT_	PA_WT_ NS1_MUT_	PA_MUT_ NS1_MUT_	PA_WT_ NS1_WT_	PA_MUT_ NS1_WT_	PA_WT_ NS1_MUT_	PA_MUT_ NS1_MUT_
Inhibition of host gene expression ^a^	+ −	− −	+ +	− +	+ −	− −	+ +	− +
Pathogenicity in mice/ Viral replication in mice lungs	+++/ +++	−/ −	−/ −	+++/ +++	++/ +	+++/ +++	+/ +	+++/ +++
Induction of innate immune response in mice ^b^/Induction of humoral responses	+++/ +++	−/ −	−/ −	+++/ +++	+/ ND	++/ ND	+/ ND	+++/ ND

^a^ PA (top) and NS1 (bottom) proteins with the ability to inhibit (+) or not inhibit (−) host gene expression. ND; Not determined. ^b^ Measured by the levels of IFN-β, CCL2, or monocyte chemotactic protein 1 (MCP-1) and TNF mRNAs in lungs from infected mice [71,174].

## Abbreviations

ACIP	Advisory Committee on Immunization Practices
ASC	Apoptosis-associated Speck-like containing a caspase-recruitment domain
ATF	Activating transcription factor
CD	Codon deoptimized
CIV	Canine influenza virus
CPSF30	Cleavage and polyadenylation specificity factor 30
CTT	C- terminal tail
dsRNA	double-stranded RNA
E1B-AP5	Adenovirus early region 1B-associated protein 5
ED	Effector domain
EIV	Equine influenza virus
eIF2α	α subunit of the eukaryotic initiation factor 2
eIF4G	Eukaryotic initiation factor 4G
FDA	Food and drug administration
HA	Hemagglutinin
IAV	Influenza A virus
IFN	Interferon
IIV	Influenza inactivated vaccine
IKK	inhibitor of kappa β kinase
IL	Interleukin
IRF	Interferon regulatory factor
ISGs	Interferon (IFN)-stimulated genes
LAIV	Live-attenuated influenza vaccine
M2	Matrix protein 2
MDV	Master donor virus
NA	Neuraminidase
NCR	Non-coding region
NEP	Nuclear export protein
NES	Nuclear export signal
NF-κB	Nuclear factor kappa β
NLRP3	NOD-like receptor family member LRR- and Pyrin domain containing-3
NLS	Nuclear localization signal
NS1	Non-structural protein 1
NSs	NS split
NXF1	Nuclear RNA export Factor 1
OAS	Oligoadenylate synthase
ORF	Open reading frame
PA	Polymerase acid
PABP	Poly(A)-binding protein
PACT	Protein activator of the IFN-induced PKR
PAMPs	Pathogen-associated molecular patterns
PB1	Polymerase basic 1
PB2	Polymerase basic 2
pH1N1	pandemic H1N1 virus
PI3K	p85-β of phosphatidylinositol 3-kinase
PKR	Protein kinase R
Pol II	Polymerase II
PR8	A/Puerto Rico/8/34
PRR	Pattern recognition receptor
RAE1	Ribonucleic Acid Export 1
RBD	RNA-binding domain
RIG-I	Retinoic acid inducible gene I
RNAse L	Ribonuclease L
RdRp	RNA-dependent RNA polymerase
ssRNA	single-stranded RNA
TNF	Tumor necrosis factor
TLRs	Toll-like receptors
TRIM25	E3 ligase tripartite motif-containing protein 25
UTR	Untranslated region
WT	Wild-type
WHO	World Health Organization
WSN	A/WSN/33
